# Developments of Plant-Based Emulsion-Type Sausage by Using Grey Oyster Mushrooms and Chickpeas

**DOI:** 10.3390/foods12081564

**Published:** 2023-04-07

**Authors:** Md. Anisur Rahman Mazumder, Naphat Sujintonniti, Pranchalee Chaum, Sunantha Ketnawa, Saroat Rawdkuen

**Affiliations:** 1 Food Science and Technology Program, School of Agro-Industry, Mae Fah Luang University, Chiang Rai 57100, Thailand; 2 Department of Food Technology and Rural Industries, Bangladesh Agricultural University, Mymensingh 2202, Bangladesh; 3 Unit of Innovative Food Packaging and Biomaterials, School of Agro-Industry, Mae Fah Luang University, Chiang Rai 57100, Thailand

**Keywords:** chickpea, consumer acceptability, hydrocolloids, mushroom, plant-based, meat alternative, protein, sausage, textural profile

## Abstract

Plant-based (PB) meat alternatives are developing due to the consumer’s demand, especially those who are mainly health-concerned. Soy proteins (SP) are commonly used as the main ingredients for PB meat analogues; however, SP may have adverse effects on the cognitive function and mood of humans. This study aimed to use grey oyster mushroom (GOM) and chickpea flour (CF) as an alternative source of SP to prepare emulsion-type sausages (ES). The effect of different hydrocolloids and oil on the quality of sausage was also investigated. The sausage was prepared using different concentrations of GOM and CF (20:20, 25:15, and 30:10 *w*/*w*). The GOM to CF ratio 25:15 was selected for the ES based on protein content, textural properties, and sensory attributes. The result indicated that sausage containing konjac powder (KP) and rice bran oil (RBO) provided a better texture and consumer acceptability. The final product showed higher protein (36%, dry basis), less cooking loss (4.08%), purge loss (3.45%), higher emulsion stability, and better consumer acceptability than the commercial sausage. The best recipe for mushroom-based ES is 25% GOM, 15% CF, 5% KP, and 5% RBO. In addition, GOM and CF could be an alternative option to replace SP in PB meat products.

## 1. Introduction

Plant-based meat substitutes are a good source of protein, and their texture, color, nutrition, and flavor can resemble particular meats [1]. Alternatives to meat made from plants are being developed in response to customer demand and the sustainability of the food supply in the future. The market for PB meat has exploded recently. According to a recent analysis by Markets and Markets^TM^, the global market for meat analogues is anticipated to be worth USD 7900 million for the year 2022 and increase to USD 15,700 million by the year 2027, with a compound annual growth rate of almost 15% in terms of value over that period [2]. There is an increase in the number of vegans and flexitarians, increased knowledge of the advantages of meat analogues over meat products, increased government funding in research and development activities, and increased investment by the food industries as key players for the quick development of PB meat analogues.

Soy protein, particularly texturized soy protein, is the prime ingredient in structured plant proteins due to its abundance, low cost, texture that resembles meat after being hydrated, and high-quality amino acid composition [3]. Soy-based meat substitutes not only have high protein and nutrition value equivalent to meat but also have little or no cholesterol and fat due to the addition of plant-based ingredients [4]. However, soy proteins have a number of drawbacks because of their anti-nutritional factors and tendency to trigger allergies [5]. Nowadays, food scientists are looking for alternative raw materials such as mushrooms, peas, and pulses to produce PB meat due to their high nutrient content, including essential amino acids, vitamins, minerals, fiber, and a certain amount of protein, as well as their low calories. These cover dietary needs as well as have a high yield and a quick planting time, and also enhance functional qualities to increase the product’s value. Growing edible mushrooms has become more and more popular as a profitable business venture, largely because of an increase in demand and market value over the past few years [6,7]. Mushrooms are a very delicious food with high protein, vitamins, and minerals, and a reduced amount of fat [8]. The *Pleurotus sajor-caju* (Fr. Singer), often known as the grey oyster mushroom, is one of the most popular mushrooms in Thailand and is grown extensively for commercial purposes year-round. In particular, GOM has abundant protein (4 g/100 g) [9] and a polysaccharide that is mostly composed of β-glucan [10]. The interaction between the protein and polysaccharide in an emulsion is very important for emulsification and gelation capacities [11]. Compared to other varieties of mushrooms, due to the unique structure of oyster mushrooms, the majority of β-glucan is water insoluble, which may result in distinct three-dimensional structural changes in the emulsion system. Chickpea (*Cicer arietinum* L.) could be another potential alternative to soy protein, due to its wide possibilities for application. One of the most frequently used and popular pulses throughout the world, it is generally sold as seeds, flour, or canned goods. In the context of alternative protein sources, chickpeas contain 17–22% dietary protein [12]. Research on the utilization of chickpea protein for meat analogues is ongoing, and the properties of chickpea protein and interaction with other ingredients in food matrices are hardly studied. Chickpea protein (CP) exhibits greater protein digestibility, a wide range of biological activity, and sufficient amounts of the essential amino acids. The mild flavor, light color, and neutral taste make them suitable for the development of new items such as noodles, breads, cookies, and sausages. Research suggests that CP and hydrolysates of CP may be used as functional ingredients for PB meat analogues [12].

Different types of hydrocolloids are widely used in the food industries due to their ability to enhance emulsion stability, increase water-holding capacity, and improve the texture and appearance of meat and meat products [13]. Different types of gums, mucilages, and water-soluble polymers are widely used as hydrocolloids which can hydrate with water to increase viscosity and form a gel [14]. It is important to consider the pH, interactions with other ingredients, viscosity, gel formation, thermo-stability, and solubility of hydrocolloids [15]. Nowadays, research on the use of different types of gum-based and protein-based hydrocolloids during the processing of meat analogues is very much necessary. Arora et al. [16] evaluated the influence of different types of binding agents (carrageenan, soy protein concentrate, casein, and xanthan gum) on the qualitative and nutritional aspects of 5% saturated fat mushroom-based sausage analogues. Carrageenan (0.8%) had the best results in terms of purge loss, cook loss, and emulsion stability, all of which enhanced process output. Moreover, sodium alginate (AL) and microbial transglutaminase (TG) are two binding substances that are frequently used in food preparation. For protein binding or gelling systems, each of these binders performs in a unique manner [17,18,19]. While TG has been used as a cold-set binder because it catalyzes covalent bonds between the ε-amino group (a primary amine) of peptide-bound lysine and the γ-carboxamide group of peptide-bound glutamine, AL has the advantage of being able to create a thermostable and irreversible gel (in the presence of calcium ion) [17,18]. The addition of konjac glucomannan (KGM) considerably altered the texture of plant-based fish balls (PFB). The hardness, chewiness, and gel strength of PFB increased dramatically with the increase in KGM. At lower levels of KGM, PFB had more and larger pores within the gel networks, resulting in a loose and weaker gel structure. The findings indicated that KGM had an excellent application in the development of plant-based seafood analogues as a key contributor to imitating the texture of seafood counterparts. By combining KGM with plant proteins, new plant-based seafood products might be developed by mimicking the texture of seafood counterparts without using complex high-pressure extrusion processes [20].

Oils and fats are important components of our diet because they serve as a vital source of energy and a carrier for vital nutrients including vitamins and bioactive chemicals. The desirable flavor, texture, and functionality of foods are provided by fats [21]. Animal fats were replaced with vegetable oils such as olive oil [17,18], palm oil [22], canola oil, and coconut oil [23,24] to lower the saturated fatty acid and cholesterol levels of certain restructured meat substitutes. Depending on the raw materials, different quantities of oil are used to provide a more meat-like texture and to improve the flavor, juicy quality, tenderness, and sensorial attributes of meat analogues. Mazlan et al. [22] utilized 10% palm oil in their soy–mushroom extrusion mixture. It has been suggested that fat acts as a lubricant in thermal-mechanical processing and helps to accelerate the development of protein alignment networks. Rice bran oil (RBO) is light, has a faint nutty flavor and is a clear, light, odorless, pale yellow liquid [25]. The World Health Organization, American Heart Association, Indian Council of Medical Research, and the Chinese Cereals and Oils Association all consider RBO to be a beneficial oil because of its fatty acid composition [26]. One of the healthiest edible oils, RBO exhibits a balanced quantity of saturated (SFA: 20% palmitic acid), monounsaturated (MUFA: 42% oleic acid), and polyunsaturated (PUFA: 32% linoleic acid) fatty acids with an average ratio of 0.6:1.1:1.0, respectively [26]. RBO typically contains 0.8% glycolipids, 1–2% phospholipids, 2–3% diacylglycerols, 1–2% monoacylglycerols, 2–6% free fatty acids, and 4% unsaponifiable fraction [27]. Compared to pork back fat (PBF) sausages, sausages prepared by RBO had the lowest cooking loss (40%). The use of RBO enhances the texture and tenderness of sausages while also substantially raising the UFA and PUFA content.

Among the food industries, the sausage industry is one of the most important sectors in the global food sector. The demand for ready-to-eat meals, such as beef sausages and hamburgers, which are thought to be the most popular meat products, has surged during the past 20 years as a result of changes in consumer lifestyles [28]. Emulsion-type sausage is made by combining fat with finely ground beef to make an emulsion and then heating it to set the structure. By using the appropriate cooking method, this product can deliver an appropriate texture and flavor that fulfills the needs of the consumer [29]. As the global sausage products demand continues to grow at a steady pace, the sausage industry is expected to flourish further. Sausage is popular due to its taste and convenience. Nonetheless, there are restrictions on the consumption of high fat and high calorie food products. The sausage industry is anticipated to continue to prosper as long as the demand for sausage products around the world grows steadily. Sausages are very popular throughout the world due to taste and convenience to carry. However, some of them are limited by the consumer due to the high fat and calorie content. Plant-based emulsion-type sausages could be an alternative option to replace meat in the current market, which requires healthy products containing high protein and low fat. Therefore, this study aimed to apply alternative meat sources for sausages that could be developed by using alternative plant-based protein sources and functional ingredients such as mushrooms and chickpeas. The objective of this study was also to determine the physicochemical characteristics such as the color, textural profile, cooking loss, proximate compositions, and protein profile (SDS-PAGE) of the developed sausages.

## 2. Materials and Methods

### 2.1. Raw Materials

Grey oyster mushrooms (*Pleurotus sajor-caju* (Fr.) Singer) were purchased from the local market in Ban Du, Chiang Rai, Thailand. Chickpea (*Cicer arietinum*) flour was purchased from Huglamool farm (Amnat Charoen, Thailand). Different sources of oils (canola oil, rice bran oil, olive oil) were purchased from the departmental store (Big C, Ban Du, Chiang Rai, Thailand). Pea protein isolate and vital wheat gluten were purchased from Union Science Co., Ltd., Chiang Mai, Thailand. Hydrocolloids (konjac powder, xanthan gum, and carrageenan) were bought from Value Industrial Products Co., Ltd. (Bangkok, Thailand). Tapioca starch and seasonings were supplied by Narah Co., Ltd. (Chiang Mai, Thailand).

### 2.2. Mushroom Preparation

The GOM was washed thoroughly using potable water twice to remove mud, dirt, and foreign materials. Extra water was removed by keeping the mushroom in a strainer for 20 min. The mushrooms were cut into 3.0 mm thickness and subjected to hot water blanching at 100 °C for 5 min. Extra water was removed by spinning through a vegetable spinner and chopped by using a meat grinder (SOKANY^®^, SK-312, Yiwu, China). The minced (coarse particle size) mushroom was dried for 10 h at 60 °C using a tray dryer (BP-80, KN Thai TwoOp, Bangkok, Thailand). The dried mushrooms were ground to a fine powder by a pulverizing machine (RT-04A, Rong Tsong Precision Technology Co., Taichung City, Taiwan). The ground mushroom was sieved through 0.500 mm (30 mesh) sized sieve [30]. Mushroom powder was stored at −20 °C using a re-sealable high density polyethylene zipper (Ziploc^®^) for further study.

### 2.3. Determination of Total Dietary Fiber (TDF) of GOM Powder

The enzymatic-gravimetric method was used to determine the TDF content of the GOM powder [31]. Defatted and dried samples were divided into two sections, which were then gelatinized and partially digested by α-amylase before being enzymatically broken down with protease and amyloglucosidase to extract the protein and starch. The GOM powder (300 g) was mixed with an acetate buffer (5 mL, 0.1 M, pH 5.0) solution containing 100 µL thermostable α-amylase and incubated at 96 °C for 60 min in a sealed tube. The sample was mixed with 400 µL of amyloglucosidase and 400 µL of protease and incubated at 60 °C for 4 h. Soluble fiber was precipitated by using 80% aqueous ethanol. Total fiber was collected through the centrifugation of samples 2000 r·min^−1^ for 20 min. The residue was then dried and weighed after being washed with ethanol and acetone. One portion of the residue was tested for protein and another for ash. By deducting the weight of the residue from the weight of the protein and ash, the TDF was calculated as a percentage of the initial sample weight.

### 2.4. Functional Properties of GOM Powder

#### 2.4.1. Water-Holding Capacity (WHC)

The WHC was determined using the methods described by Mesías and Morales [32]. First, 5 g of flour was inserted in a pre-weighed centrifuge tube with 30 mL of water. The mixture was vortexed rapidly for 1 min, and then kept at 25 °C for 30 min, before being centrifuged at 3500 rpm for 15 min. The non-absorbed water was removed, and the tube was weighed. The results are expressed as g of water retained per 100 g of flour. The WHC was calculated using the following Formula (1):

(1) $$\mathrm{WHC}\left(g/g\right)=\frac{X-Y}{Z}$$

where X = weight of tube with sample and water retained (g); Y = weight of tube with sample (g); Z = weight of sample (g).

#### 2.4.2. Oil-Holding Capacity (OHC)

The OHC was determined by using methods described by Sosulski et al. [33]. One g of GOM powder was placed in a centrifuge tube and weighed. Ten ml of soybean oil was added to the centrifuge tube, and the GOM oil was mixed properly. The mixture rested for 30 min, and was centrifuged at 2500 rpm for 20 min. The oil was removed, and the weight of the centrifuge tube was calculated. The oil-holding capacity was calculated as follows (2):

(2) $$\mathrm{OHC}\left(g/g\right)=\frac{\mathrm{Weight}\;\mathrm{of}\;\mathrm{sample}\;\mathrm{after}\;\mathrm{centrifuge}\;\left(g\right)-\mathrm{Weight}\;\mathrm{of}\;\mathrm{sample}\;\mathrm{before}\;\mathrm{centrifuge}\;\left(g\right)}{\mathrm{Weight}\;\mathrm{of}\;\mathrm{sample}\;\left(g\right)}$$

#### 2.4.3. Swelling Capacity (SC)

The SC of the GOM powder was determined using the methods described by Elaveniya and Jayamuthunagai [34]. A pre-weighed 50 mL centrifuge tube was filled with 1 g of GOM powder, and 10 mL of distilled water was added to the tube. The centrifuge tubes were heated in a shaking water bath at 80 °C for 30 min (Schufzart, Membart GmBH Co., Bchenbach, Germany). After removing the centrifuged tubes from the water bath, they were dried and cooled at 25 °C. Finally, the solution was centrifuged at 2000 rpm for 15 min, and the supernatant was evaporated. The SC was determined by decanting the supernatant and weighed.

(3) $$\mathrm{SC}\left(g/g\right)=\frac{\mathrm{Weight}\;\mathrm{of}\;\mathrm{wet}\;\mathrm{mass}\;\mathrm{in}\;\mathrm{sediment}\;\left(g\right)}{\mathrm{Weight}\;\mathrm{of}\;\mathrm{dreid}\;\mathrm{matter}\;\mathrm{in}\;\mathrm{gel}\;\left(g\right)}$$

#### 2.4.4. Emulsion Activity (EA) and Emulsion Stability (ES)

The EA was determined according to Sathe et al. [35] with the following modifications. First, 100 mL of 2% (*w/v*) protein suspension at different pHs (ranging from 2 to 10) was homogenized at 10,000 rpm for 30 s using a homogenizer (T25 digital ULTRA-TURRAX^®^, Staufen, Germany). Corn oil (100 mL) was added and homogenized for 1 min. The emulsions were centrifuged at 3000 rpm for 5 min, and the volume of emulsion remaining was determined using the following Formula (4).

(4) $$\mathrm{EA}\left(\%\right)=\frac{\mathrm{Volume}\;\mathrm{of}\;\mathrm{emulsified}\;\mathrm{layer}}{\mathrm{Volume}\;\mathrm{of}\;\mathrm{whole}\;\mathrm{layer}\;\mathrm{in}\;\mathrm{centrifugetube}}\times 100$$

Emulsions prepared using the aforementioned procedures were heated at 80 °C for 30 min, and cooled at 25 °C. The emulsion was centrifuged at 3000 rpm for 5 min to measure the stability of the GOM powder and CF. The ES was calculated using the following Formula (5):

(5) $$\mathrm{ES}\left(\%\right)=\frac{\mathrm{Volume}\;\mathrm{of}\;\mathrm{remaining}\;\mathrm{emulsified}\;\mathrm{layer}}{\mathrm{Original}\;\mathrm{volume}\;\mathrm{of}\;\mathrm{emulsion}}\times 100$$

### 2.5. Processing of Emulsion-Type Sausage (ES)

In order to develop an ES, there were four experimental designs as follows:

(i) To find the suitable concentration of GOM powder, and CF for the processing of ES, the following ratios of GOM powder and CF were used: 20:20, 25:15, 30:10 (*w*/*w*). Other ingredients, e.g., vital wheat gluten (15% *w*/*w*), canola oil (5% *w*/*w*), wheat flour (5% *w*/*w*), pea protein isolate (5% *w*/*w*), seasoning (5% *w*/*w*), tapioca starch (10% *w*/*w*), konjac powder (5% *w*/*w*), and water were added to the mixture (Table 1). To prepare the batter for the sausage, first, different concentrations of GOM powder and CF were mixed properly in a mixer machine (5KPM5EWH, KITCHENAID^®^, St. Joseph, MI, USA) at a speed of 150 rpm. Thereafter, 50% of other ingredients were added to the mixer machine and mixed properly. The rest of the 50% ingredients were added to the mixer machine and mixed for 10 min. The batter was stuffed into cellulose casings and steamed for 10 min at 100 °C, followed by cooling at 25 °C. For the suitable formula, a concentration of GOM powder and CF was chosen based on the texture profile analysis (TPA), protein content, and consumer acceptability. The developed ES was compared with the commercial plant-based sausage.
(ii) To find the suitable hydrocolloids (5% *w*/*w*), konjac powder (KP), xanthan gum (XG), and carrageenan (CN) were used in this study to quantify the quality attributes of the sausage. To find the suitable formulation, the developed sausages were compared with commercial sausages based on the textural profile analysis and sensory attributes.
(iii) To find the suitable source of edible oil (5% *w*/*w*), rice bran oil (RBO), olive oil (OL), and canola oil (CO) were used to find the quality attributes of the sausage. To find the suitable formulation concentration of GOM powder, CF, type of hydrocolloids, and sources of oil, the formulated sausages were subjected to a sensory analysis by panelists regarding appearance and taste.

**Figure 1:**
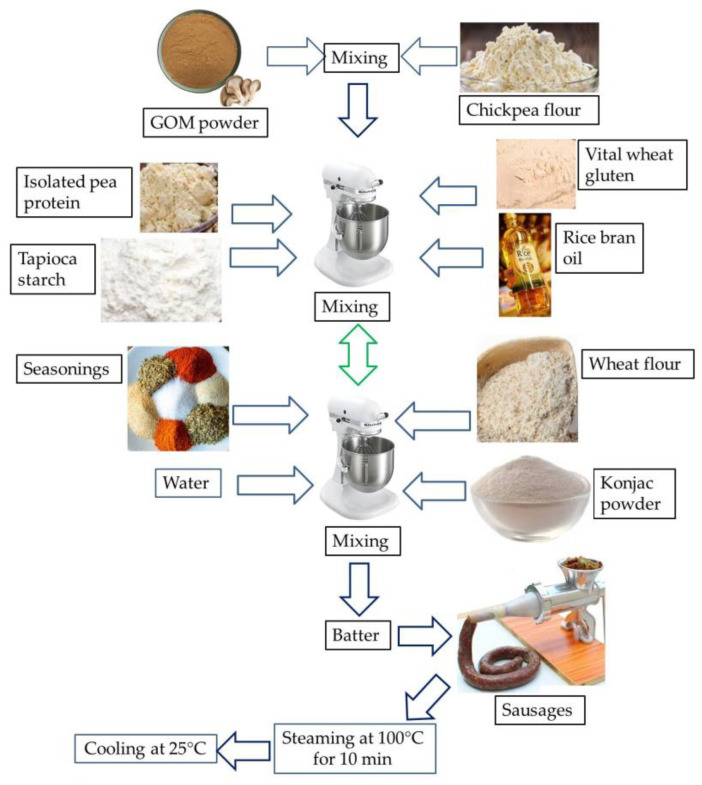
Processing of grey oyster mushroom-based emulsion-type sausages.

The final ESs were prepared using GOM powder 25% (*w*/*w*), CF (15% *w*/*w*), vital wheat gluten (15% *w*/*w*), rice bran oil (5% *w*/*w*), wheat flour (5% *w*/*w*), pea protein isolate (5% *w*/*w*), seasoning (5% *w*/*w*), tapioca starch (10% *w*/*w*), konjac powder as hydrocolloids (5% *w*/*w*), and water (15% *w*/*w*). Figure 1 shows the flow diagram for the processing of the ESs.

### 2.6. Determination of Physical Characteristics of GOM-Based Emulsion-Type Sausages

#### 2.6.1. Texture Profile Analysis (TPA)

A TPA of GOM-based ES was determined using the Aguirre et al. [36] methods with slight modifications. The ES was cut into 10 × 2.0 × 3.0 cm^3^ (length × width × height). The TPA was measured using a TA.XT plus texture analyzer (Stable Micro Systems, Surrey, UK), and a cylindrical probe with 3.6 cm diameter, 50% compression, and a test speed of 1.0 mm/s was used. The textural characteristics were assessed using 2-cycle compression with a 50 kg load cell and cross-head velocity of 100 mm/s. The following parameters were used: pre-test speed 2.0 mm/s; post-test speed 2.0 mm/s with 80% compression. Hardness, chewiness, gumminess, cohesiveness, and springiness were determined for the emulsion-type sausages. The TPA of the developed GOM-based ES was determined using Texture Expert 1.05 software (Stable Microsystems, Godalming, UK).

#### 2.6.2. Expressible Moisture

The GOM-based ESs with width × length × height (0.5 × 1.0 × 0.5) cm^3^ were placed between the Whatman filter paper (no. 4). Whatman filter paper (5-piece) was used to absorb the expressible water during pressing. The sheet was pressed with a constant force of 10 kg/cm for 2 min. The weight of the developed ES was taken before and after pressing, and the percentage of moisture excreted after pressing was calculated (6) [37].


(6) $$\mathrm{Expressible}\;\mathrm{moisture}\left(\%\right)=\frac{\mathrm{Weight}\;\mathrm{before}\;\mathrm{pushing}\;\left(g\right)-\mathrm{Weight}\;\mathrm{after}\;\mathrm{pushing}\;\left(g\right)}{\mathrm{Weight}\;\mathrm{before}\;\mathrm{pushing}\left(g\right)}\times 100$$

#### 2.6.3. Emulsion Stability

The capacity of an emulsion to resist changes over time is referred to as emulsion stability. Emulsion stability was determined before filling the batter into the casings using a modified method by Hughes et al. [38]. A 15 g sample was centrifuged for 1 min at 7000 rpm in a centrifuge (Sorvall^TM^ ST 8 Small Benchtop Centrifuge, Thermo Fisher Scientific, Waltham, Massachusetts, USA). The sample was then heated at 70 °C for 30 min and centrifuged again at 7000 r·min^−1^ for 3 min. A pre-weighed crucible was filled with the supernatant, and the weight was recorded. The sum of the water and fat exudates was used to calculate the total exudate. The ratio between the weights of the supernatant to the weight of the sample was used to calculate the total exudate and convert it into the percentage.

#### 2.6.4. Purge Loss

Prior to steam processing, 10 g fresh GOM-based ESs were weighed together and stored at −20 °C for 2 weeks. Frozen sausages were subjected to thawing at 28 ± 2 °C for 6 h and weighted again. The ratio between the weights before and after thawing was calculated and converted into a percentage.

#### 2.6.5. Cooking Loss

The cooking loss was determined using four different ESs by the percentage weight difference of the sausages before and after cooking. The sausages were cooked at low flame heat for 5 min and cooled at 28 ± 2 °C. The cooking loss was calculated using the following Equation (7) [39]:

(7) $$\mathrm{Cooking}\;\mathrm{loss}\left(\%\right)=\frac{\mathrm{Weight}\;\mathrm{of}\;\mathrm{raw}\;\mathrm{sausages}\;\left(g\right)-\mathrm{Weight}\;\mathrm{cooked}\;\mathrm{sausages}\;\left(g\right)}{\mathrm{Weight}\;\mathrm{of}\;\mathrm{raw}\;\mathrm{sausages}\;\left(g\right)}\times 100$$

#### 2.6.6. Color Determination

The color of the GOM-based ES was determined by using a colorimeter (Hunter Lab/colorQuest XE, Reston, Color Global, Bangkok, Thailand) (10° standard observers, illuminant D65, Hunter Associates Laboratory: Reston, VA, USA) and calibrated with a standard white plate. The CIE L*, a*, and b* values of the samples were recorded using ten randomly chosen samples. The L* value ranged from 0 to 100. The luminance (L* value) ranged from total darkness (L* = 0) to white (L* = 100). The a* value indicated red for a positive value and green for a negative value. The yellow b* value was positive and blue was the negative b* value. These attributes were also used to determine E and whiteness, according to Lee et al. [40]. A ΔE > 2.0 was often considered to be a color difference. Based on triplicate measurements, the mean color values were calculated.

### 2.7. Determination of Chemical Characteristics of GOM-Based Emulsion-Type Sausages

#### 2.7.1. Proximate Composition Analysis

Moisture, ash, protein, and fat content were calculated using the 2019 AOAC guidelines. Moisture content was calculated by putting 3–5 g of the sample into a hot air oven for 16 h at 105 °C [41]. Ash content was determined using a muffle furnace for 6 h at 525 °C [42]. The Kjeldahl method, which uses a nitrogen-to-protein conversion factor of 5.99, was used to determine the protein level [43]. Fat content was measured using the Soxhlet extraction method [44]. The amount of total carbohydrates was calculated in accordance with the FAO guidelines [45].

#### 2.7.2. SDS-Polyacrylamide Gel Electrophoresis (SDS-PAGE)

Meat sausage, commercial plant-based sausages, and ES were analyzed for protein patterns using SDS-PAGE. The samples were mixed at a 1:1 ratio with a sample buffer (0.5 M Tris-HCl, pH 6.8, 4% SDS, 20% glycerol, 0.03% bromophenol blue with/without 10% DTT), and boiled for 3 min. The protein-dyed samples were put onto the Roti-PAGE Gradient (4–20%) pre-cast gels and run in a buffer solution-filled electrophoresis tank utilizing a Biostep^®^ GmbH power supply (Burkhardtsdorf, Germany) at a constant current of 60 mA. The gels were stained in a staining solution (Coomassie Blue R-250 methanol-acetic acid) followed by gentle shaking at 50 rpm overnight. The gel was de-stained with de-staining solutions I and II (methanol-acetic acid-water) until the background was clean and then dried.

### 2.8. Sensory Evaluation

The sensory evaluation of the sausages was carried out using a 9-point hedonic scale (where 1 = dislike extremely, and 9 = like extremely) [46]. The sensory analysis was conducted at the Food Sensory Lab (S4), Mae Fah Luang University, Chiang Rai, Thailand. Ethical approval of the consumer testing and evaluation of the products (Protocol No.: EC22177-14) was permitted by Mae Fah Luang University, Chiang Rai, Thailand (COE. 168/2022). Samples were evaluated by at least 30 untrained panelists. Each study of GOM-based ES was evaluated based on its sensory attributes, which included appearance, aroma, texture, and overall acceptability. All the panelists were also requested to provide their suggestions and opinions about the development of the product.

### 2.9. Statistical Analysis

Each data analysis was performed in triplicate, and the mean standard deviation was utilized to calculate the results. An analysis of variance was performed to assess various formulations by the Statistical Tool for Agricultural Research (STAR) software (International Rice Research Institute, Manila, The Philippines). The significance level of *p* < 0.05 was used to determine whether the two groups were statistically different.

## 3. Results and Discussion

Table 2 shows the nutritional composition of the GOM powder and CF. Table 2 suggests that GOM powder has a high concentration of protein (23.10% vs. 22.05%), ash (7.15% vs. 2.72%), and dietary fiber (55.12% vs. 10.80%), but lower levels of fat (2.52% vs. 4.61%), moisture (6.92% vs. 7.95%), and carbohydrates (60.31% vs. 62.62%) than CF. GOM powder contains significant amounts of TDF, indicating that mushroom fruiting bodies are rich in TDF. High TDF-containing GOM powder might be a great alternative to refined powder or other conventional TDF sources for the processing of healthy products.

Functional properties are related to the physical and chemical structure of plant polysaccharides. Table 2 shows the functional properties of GOM powder and CF. All of the functional properties were higher in GOM powder than in CF. The functional properties of powder products depend on the hydrophilic constituents, including polysaccharides and proteins, which are connected to diffusion processes and water affinity [47]. The WHC is the amount of water that is firmly attached to the hydrated fiber following the application of external centrifugal gravity or compression [48]. A high WHC (12.75 g/g) suggests that GOM powder has prospective uses in food products for viscosity development and freshness retention. Higher WHC, OHC, and SC of the GOM powder were probably due to the presence of higher dietary fiber (55.12%) and the porous morphology favored the absorption, retention, and swelling of flour particles in water. GOM powder shows a higher WHC than CF. High WHC flours may be beneficial in food product applications such as bakery formulation and processing of meat analogues due to the absorption ability of the proper quantity of water into the dough, improving handling characteristics, and keeping freshness [49]. Nonetheless, dietary fiber in GOM powder has the ability to adsorb water inside the fiber matrix, preventing the degradation of the structure. The high WHC of GOM powder may be explained by the high TDF concentration [50,51]. The capacity of dietary fiber to increase bulk after absorbing water is known as the SC and is quantified as settled bed volume [52]. The SC was higher in GOM powder than CF (Table 2). The OHC is also a technological property associated with the chemical structure of plant polysaccharides that is influenced by the thickness, surface characteristics, overall charge density, and hydrophobicity of the fiber particle [53]. The capacity of GOM powder to retain oil is very crucial in the food industry, such as minimizing oil losses while cooking. The high OHC of GOM powder might be due to the high TDF that can absorb oil [48]. Kaushal et al. [54] suggested that more hydrophobic proteins have stronger lipid-binding, indicating that non-polar amino acid side chains bind the paraffin chains of the lipids. The OHC is necessary since fats function as a flavor keeper and improve the mouth feel of meals [55]. This research shows that GOM powder had a higher OHC than CF, suggesting that CF (OHC, 3.97 mL/g) has less accessible non-polar side chains in its protein molecules than GOM powder, which has a higher OHC (7.82 mL/g). In addition to the chemical factors, the physical aspects (drying and grinding the GOM into powder) increased the surface area and improved absorption, leading to significant WHC, SWC, and OHC [51]. The EA may be described as the ability of a molecule to aid in the solubilization or dispersion of two immiscible liquids. The capacity to preserve the integrity of the emulsion is termed emulsion stability. The GOM powder had higher EA (50.55%) and emulsion stability (96.10%) than CF (EA, 36.44% and emulsion stability, 90.15%) and may be utilized in food products to generate emulsions and extend the shelf life. The EA and emulsion stability of the GOM powder may be due to the high protein content of GOM powder as proteins are known as powerful emulsifying agents [53].

### 3.1. Effect of Concentration of GOM Powder and CF on the Protein, TPA, and Sensory Quality of ES

The protein content of commercial PB sausages (C) and GOM-based ESs are shown in Table 3. Commercial PB sausages were made exclusively from hemp and soy, and 50% of them were soy protein. The result suggests that the protein content was higher (*p <* 0.05) in all GOM-based ESs than in the control PB sausages. The protein content was higher in GOM-based ESs for several reasons: (i) edible mushrooms contain various bioactive components such as proteins (a certain amount of protein around 4 g/100 g) [9]; (ii) chickpeas are a rich source of dietary proteins (17–22%) when compared to other protein sources [12]; and (iii) pea protein isolate, where the protein content = 23 g/100 g, which is about 38.3% of the total nutrients [56]. Of the fifteen isolates studied, the pea protein isolate (PPI) had the third-highest protein content percentage (81%)—just below wheat protein and caseinate protein. However, there was a significant difference (*p <* 0.05) between the three developed sausages based on protein content. Increasing the concentration of CF significantly (*p <* 0.05) increased the protein content of the sausages (Table 3). Dietary protein, for example, is essential for functional needs such as improving health, muscle, and growth [57]. The recommended dietary allowance (RDA) for protein may be readily met by the consumption of GOM-based ES. The protein content in 100 g GOM-based ES showed higher protein content than in C. As the commercial sausages were made exclusively from hemp and soy, the developed ES sausage contained more protein than the commercial options [56]. In comparison to lean pork, the GOM-based ESs enhanced with chickpea flour, pea protein isolate, and wheat flour would provide more than 65% of the RDA for protein, according to the Institute of Medicine, Food and Nutrition Board [58] RDA for daily dietary protein intake for an adult aged 19–50 years reported in 2001.

Research on the physicochemical and nutritional qualities of chickpeas has shown that it might be feasible to replace the meat in nuggets and sausage types of products [59,60,61]. Moreover, for food industry applications, chickpeas are converted into chickpea flour to increase their functionality. Nowadays, CF is only used as meat binders and extenders in meals, despite being able to be utilized as a meat alternative due to its high protein content [62]. According to many researchers, CP or CF is utilized in the processing of meat analogs as well as in improving the texture of various meat products and plant-based meat alternatives. For instance, the addition of CF at 10 to 20% produces the ideal protein to starch ratio, enabling adequate protein texturization and the processing of product qualities that may potentially mimic meat [63]. Two major proteins in CF are albumin and globulin. The techno-fractional properties of proteins demonstrate that chickpea protein may be used in the technology of gluten-free, low-gluten food processing. Without gluten, things do not stick together smoothly. Due to the inherent density and unique binding properties, CF is naturally dense flour. When mixed with other gluten-free flours, CF produces a thick but soft texture [64]. Modern gluten-free and low allergen PB meat analogs with better textural, nutritional, and organoleptic qualities are made with CF/CP [65]. Overall, the mixing of GOM powder with different concentrations of CF may produce a product with the desired textural qualities. 

#### 3.1.1. Texture Profile Analysis of GOM-Based ES

The TPA of the developed GOM-based ES is shown in Table 3. The developed GOM-based ESs showed significant differences (*p <* 0.05) in terms of hardness (5028.14–5485.79 N) and springiness (0.79–0.90 mm) from the commercial PB sausages. The findings showed that, in comparison to the commercial PB sausages, the GOM-based ES had a firm texture and required a lot of elasticity force. This study suggested that MR25 (25% grey oyster mushroom, and 15% chickpea flour) showed a comparable texture to the commercial PB sausages. The major ingredients of commercial sausages are hemp and soy protein, which contribute to the product’s hardness. In this investigation, the concentration of CF and GOM powder both consistently enhanced the hardness of the developed sausages. Increasing the CF and decreasing the GOM powder increased the hardness of the sausages. The protein content was increased by increasing the concentration of CF. However, the addition of the grey oyster mushroom up to 50% in chicken patties by itself obtained a softer texture compared to the addition of 25% grey oyster mushroom in chicken patties [65]. The changes in hardness in sausages may be explained by the molecular structure of the protein. The lower hardness can be explained from a molecular level. A lower presence of cysteine amino acid residues resulted in a lower cross-linking, leading to a higher porous structure with a high water-holding capacity and therefore a softer texture [66]. Not only did the presence of a high-concentration protein allow for interactions for cross-linking, but the presence of less starch also meant that less interfered with the protein–protein interactions [63]. Though chickpea flour contains a sufficient amount of starch (51.2 ± 0.26 g/100 g) [67], it was not able to retain sufficient water molecules to soften the texture due to the gelatinization of starch during heat treatment. CF is a strong source of fiber (4 to11%), but less than the grey oyster mushroom (17%). Increasing the fiber content increases the water-holding capacity and softens the texture of sausages. 

Chewiness, springiness, and cohesiveness are also important parameters for the evaluation of a product’s texture. In contrast to springiness, which describes how well a product physically returns to its original condition following the first compression [68], chewiness is defined as the amount of energy needed to chew a meat product [69]. Cohesiveness is connected to the extent to which food and food products may be deformed before they rupture [69]. In this investigation, it was suggested that with the exception of MR25, the springiness of commercial sausages was significantly (*p <* 0.05) higher than that of the developed ES. The statistical analysis suggested that there were no significant differences (*p* > 0.05) between the commercial and developed samples; even all three developed ESs did not show any significant differences (*p >* 0.05) in terms of chewiness and cohesiveness, respectively. However, Sharima-Abdullah et al. [65] found that the chewiness of imitation chicken nuggets was consistently decreased when the chickpea flour increased (grey oyster mushroom stems decreased). Wan Rosli et al. [70] showed that as the amount of grey oyster mushroom increased, the cohesiveness of the chicken patty reduced significantly.

#### 3.1.2. Sensory Evaluation

The sensory analysis suggested that MR25 showed better acceptability according to the 30 untrained panelists. There were no significant differences (*p >* 0.05) between all three samples in terms of texture and taste. However, overall acceptability showed significant differences among all three samples. Though protein content was higher in MR20, based on the TPA and sensory analysis, MR25 (25% *w*/*w* GOM powder and 15% *w*/*w* CF) was selected for the next experiments and for the final ES processing.

### 3.2. The Effect of Different Hydrocolloids on Quality Attributes of GOM-Based ES

#### 3.2.1. Effect of Hydrocolloids on Emulsion Stability of ES

The impacts of KP, XN, and CN at 5% *w*/*w* on the emulsion stability of GOM-based ES are shown in Table 4. The total exudate (%) for GOM-based ESs prepared with three types of additives, namely, KP, CN, and XN ranged from 2.30 to 2.87. Emulsion stability was inversely correlated with the total exudates. KP at 5% *w*/*w* demonstrated the least amount of total exudate (2.30%) and so gave ES the best emulsion stability, followed by CN (5% *w*/*w*) and XN (5% *w*/*w*), and a total exudate of 3.68 and 4.87%, respectively. Konjac gum is the main ingredient of konjac powder, sometimes referred to as Konjac Glucomannan (KGM). It is a kind of dietary fiber purified from konjac flour as a raw material. It is entirely made up of soluble fiber and is free of starch, sugar, protein, and fat. Other physical and chemical characteristics of glucomannan powder include water solubility, preservation, thickening, stability, suspension, gelation, and adhesion. The distinguishing quality of konjac glucomannan fiber is its exceptional capacity to gel, which manifests as the formation of an elastic, stable, and irreversible gel [71]. Konjac extract is not only a natural food additive but is also known as a healthy food additive. It is one of the best foods for human health in the twenty-first century, according to the World Health Organization. The two-way ANOVA indicated that there was a significant difference (*p <* 0.05) among all three samples in terms of total exudate (%). However, the stability of the batter emulsion was enhanced by the addition of all the additives. This might be explained by the fact that these chemicals can form a gel, which leads to better water and fat retention during the processing of sausages. XN demonstrated the least emulsion stability, while KP and CN demonstrated the improved emulsion stability of sausages. However, Hughes et al. [38] observed that the addition of carrageenan significantly improved the emulsion stability of frankfurter sausages. In low-fat beef frankfurters, an increase in carrageenan concentrations increased the emulsion stability [72].

#### 3.2.2. Effect of Hydrocolloids on Purge Loss of ES

The least amount of purge loss (3.25%) was seen in the GOM-based ES utilizing KP (5% *w*/*w*) as a binding agent after being frozen, as compared to other additives (Table 4). The purge loss percent ranged from 3.25 (KP at 5% *w*/*w*) to 5.05% (XN at 5% *w*/*w*). There was a significant difference between each of the three samples at the 5% level of significance based on the sources of the additives. Reduced purge loss with the addition of binding agents is responsible for improvements in water binding and hydration properties that lead to a decrease in drip losses during thawing. Based on purge loss, KP exhibits greater emulsion stability due to the exceptional capacity for gel formation, which is an elastic, stable, and irreversible gel [71]. However, Shand et al. [73] discovered that adding carrageenan to beef rolls at levels of 0.5 and 1% decreased purge loss during refrigerated storage.

#### 3.2.3. Effect of Hydrocolloids on the Cooking Qualities of ES

The results of cooking loss on the GOM-based ES are shown in Table 4. The addition of different additives showed differences (*p <* 0.05) in the cooking loss. The best results were produced by KP, followed by CN and XN. The sample with XN had the highest cooking loss (CL, 3.24%), while the sample with KP had the lowest CL (1.44%). CL is connected to a number of different aspects, including the structure of the protein network, the type of protein, the water-holding capacity of the proteins, and the fiber content [74]. A confined compression test revealed a relationship between the pore structure of meat substitutes and cooking loss [75]. These holes may allow water to easily escape from the meat analogues [76,77]. KP is a kind of dietary fiber, entirely made up of soluble fiber and is free of starch, sugar, protein, and fat. It has better water solubility, thickening, stability, suspension, gelation, and adhesion capacity. KP shows exceptional capacity to gel, which manifests as the formation of an elastic, stable, and irreversible gel [76]. Kojac powder shows the best results in reducing purge loss, cooking loss, and increasing emulsion stability, and thereby it will improve the overall process yield. However, the addition of all three additives reduces the CL. The addition of gums significantly reduced the CL for sausages observed by Pietrasik and Duda [78] and Carballo et al. [79]. Similarly, CL was decreased for frankfurters prepared with peach dietary fiber [80,81]. Russell Egbert et al. [82] and Candogan and Kolsarici [83] found that cellulose and carrageenan could lower the cooking losses. 

This study assessed the expressible moisture (EM, %) during compression of the cooked samples in order to acquire insight into the moisture loss of the various samples. EM (%) was significantly (*p <* 0.05) lower in KP sausages and higher in XN- and CN-processed sausages (CN, 0.58 ± 0.06%; XN, 0.61 ± 0.02%). There were no significant differences (*p >* 0.05) between XN- and CN-processed ES based on EM. Moreover, processed ES from XN (6.97%) and CN (5.79%) had the highest CL. Thus, they were still able to release more fluid during compression; even more fluid had previously been released during cooking. This could be connected to particular structural changes in the tissues of sausages. Consequently, even though significant changes in CL were seen, EM showed only minor variability. This may suggest that the initial moisture content is not related to EM, but EM is only influenced by protein concentration, type, and structure. According to several studies [77,84,85], heat exposure during the processing of meat analogs causes structural changes that promote a better water-holding capacity. This might account for the reduced EM in KP sausages. 

#### 3.2.4. Effect of Hydrocolloids on Textural Properties of ES

The TPA of all ESs, including hardness, springiness, cohesiveness, chewiness, and gumminess made from konjac powder, xanthan gum, and carrageenan, were not significantly different (*p >* 0.05) (Table 4). Research suggests that hydrocolloids increase the water-holding and retention capacity of meat analogs [86]. Nevertheless, the textural properties of beef products are influenced by moisture and fat content, which have an effect on the water-holding capacity and emulsion stability [87]. In general, hydrocolloids increase the capacity to store and bind water, which results in slightly lowered textural qualities. Research revealed that restructured duck hams without hydrocolloids showed lower springiness than treated ones. The textural characteristics of beef products were enhanced with xanthan gum and carrageenan [88]. Dai et al. [89] found that the inclusion of hydrocolloids resulted in lower TPA ratings for all of the evaluated meat items compared to the control sample (*p <* 0.05). In general, alginate and calcium ions can form a gel [90]. Alginate’s ability to form a gel was limited and its hardness was lower than that of other treatments when it was coupled with CN and KP (*p <* 0.05) in the duck ham, due to a lack of calcium ions in the duck. The combination of carrageenan and konjac with alginate in duck ham can improve the hardness, cohesiveness, gumminess, and chewiness. Nevertheless, meat products with added hydrocolloids may have a meat product with a soft texture [20].

#### 3.2.5. Effect of Hydrocolloids on Sensory Attributes of ES

The sensory analysis suggested that 5% *w*/*w* of konjac powder formula was the most suitable for the processing of ES. Though, the overall acceptability of all three types of emulsion-type sausage was not significantly different (*p >* 0.05) from each other. The emulsion-type sausage with KP was selected for the further step due to the consumer acceptance of 40% of it as is demonstrated in Table 4. According to results reported by Ran et al. [20], the inclusion of konjac glucomannan had a substantial impact on the TPA of plant-based fish balls. The addition of 5% KP fish balls (plant-based) dramatically increased their TPA and gel strength [20]. According to Park et al. [91], adding hydrocolloids to pork sausages changed their taste. Their result suggested that the addition of hydrocolloids to meat products appears to have a generally favorable impact on their sensory qualities.

### 3.3. The Effect of Oil on Quality Attributes of GOM-Based ES

The sensory analysis indicated that consumers accept RBO as the most suitable oil for the processing of GOM-based emulsion-type sausages. However, the overall acceptability of all three types of ES was not significantly different (*p >* 0.05) from each other (Table 5). The emulsion-type sausage with RBO was selected for the further step due to the consumer acceptance of 43.3%, which is demonstrated in Table 5. There was a significant difference (*p <* 0.05) between RBO and OL in terms of appearances.

RBO is a great alternative to animal fats because of its superior physicochemical qualities, neutral flavor, and antioxidant content [92]. Compared to the control, the hardness decreased with the increase in RBO in the sausage [93]. Similarly, Ahmed et al. [94] found that the addition of vegetable oils to replace animal fats increased the hardness of hamburgers. Vegetable oils may have an advantage in this impact due to having fewer fat globules than animal fats, which leads to more protein–protein and protein–lipid interactions. According to Cîrstea et al. [95], replacing back fat with pre-emulsified plant oils led to the development of water- and fat-binding characteristics, reducing the fluid loss in chicken liver paste. These findings suggested that RBO might stabilize oil/water emulsions and that utilizing pre-emulsified meat batters instead of adding RBO directly to the emulsion generated firmer emulsion-type pork sausages. As a result, RBO in place of PBF can be utilized to produce sausages with a softer texture. These findings were crucial for the sausages’ sensory qualities [96].

### 3.4. Analysis of GOM-Based ES

The GOM-based ES was designed based on 25% *w*/*w* GOM powder, 15% *w*/*w* CF, 15% *w*/*w* vital wheat gluten, 5% *w*/*w* RBO, 5% *w*/*w* wheat flour, 5% *w*/*w* pea protein isolate, 5% *w*/*w* seasoning, 10% *w*/*w* tapioca starch and 5% *w*/*w* konjac powder as hydrocolloids, and 15% *w*/*w* water. The GOM-based ES was further analyzed for sensory attributes; physicochemical, nutritional, and textural properties; and emulsion stability.

#### 3.4.1. Sensory Attributes of GOM-Based ES

This study compared ES and C sausages on the basis of sensory attributes. This study used 30 untrained panelists using a nine-point hedonic scale to find statistical differences (Table 6). Taste, appearance, texture, and overall acceptability were determined. According to a one-way ANOVA, there were no discernible differences between the commercial© and developed ESs. Thus, the result concluded that with 95% confidence, there is not enough evidence to differentiate ES and C sausages. The processed GOM-based ES might be similar to the commercial plant-based sausages in terms of sensory attributes.

#### 3.4.2. Physical and Chemical Properties of GOM-Based ES

Chemical Properties

The proximate analyses of ES on a dry weight basis (g/100 g) are displayed in Table 7. ES contained significantly (*p <* 0.05) higher amounts of protein than C sausages. Dietary protein is necessary for functional demands including improving health, building muscle, and promoting growth [57]. GOM-based ESs may be consumed to easily meet the recommended dietary intake (RDA) for protein. Protein content in 100 g ES will supply more than 65% of the RDA for protein compared to lean pork based on the Institute of Medicine, Food and Nutrition Board’s [58] RDA for daily dietary protein consumption for an adult aged 19 to 50 years (reported in 2001). These ESs will contribute 9 to 16% of fat for men and 11 to 19% of fat for women, based on the Institute of Medicine, Food and Nutrition Board’s [58] RDA for daily fat intake for an adult aged 19–50 years reported in 2001. There was no significant (*p >* 0.05) difference between C and ES in terms of moisture, and ash content. Nonetheless, ES showed higher protein and less fat content than C sausages. Both ES and C sausages showed low energy content, which is very good for healthy consumers.

##### Physical Properties

The TPA measures several texture parameters including hardness, cohesiveness, gumminess, springiness, and chewiness [36]. The term hardness describes the sausage’s maximal resistance to being bitten by teeth. The ability of a sausage to maintain its structural integrity is determined by its cohesiveness. The ability of the sausage to recover from deformation defines its springiness and resilience, while the effort needed to chew the meat products is what defines gumminess (the combination of hardness and cohesiveness) and chewiness (the combination of gumminess and springiness). Table 7 provides a summary of the TPA of ES. The amount of CF and GOM powder in the ES was associated with an increase in hardness, cohesiveness, chewiness, springiness, and gumminess. Similar trends were reported by Zeraatkar et al. [97] who found that the hardness of chicken sausage increased when meat was partially or entirely substituted by plant-based protein. That could be due to chickpea and mushroom supplements in varying amounts and dosages. The addition of only 5% of pea protein isolates in the mushroom and chickpea mixture contributed to increased hardness. The TPA indicated that there was a significant difference (*p <* 0.05) between ES and C sausages in terms of hardness, gumminess, and springiness. All three parameters were higher in ES, but chewiness and cohesiveness did not show any differences (*p >* 0.05) for ES and C sausages.

The color of cooked ES showed L* (lightness) = 42.6 ± 0.45, a* (13.52 ± 0.33) and b* (20.98 ± 0.73), which were not significantly different (*p >* 0.05) from commercial PB sausages (Table 7). In general, the color of food products is the effect of colored natural products which are linked to raw materials and/or colored compounds generated during processing [98]. The L* value measures luminosity and reflects the black-white component. A decrease in L* values suggests the darkening of products.

Cooking loss, emulsion stability (measured as total extrudate), and purge loss were higher (*p <* 0.05) in C sausages than in ES (Table 7). This might be due to the differences in the protein sources, types of oil, and additives. The reduced cooking loss in ES may be attributed in part to the dietary fiber from chickpea, konjac powder, and mushrooms. Moreover, Petracci et al. [99] observed that the inclusion of sugarcane fiber decreased the cooking loss, water loss, and fat loss of chicken sausages. Dietary fiber physically absorbs water, alters the pH value of systems, stabilizes lipids, and improves the combination of proteins, lipids, and other carbohydrates to increase the cooking yield and emulsion stability of meat products [100]. Nutritional, physical, sensory, and textural analyses suggested that ES can be a vital source of nutrition and may be successfully introduced into the market. However, bioavailability and in vivo analyses are further required to determine successful implications in the market.

### 3.5. Protein Patterns

**Figure 2:**
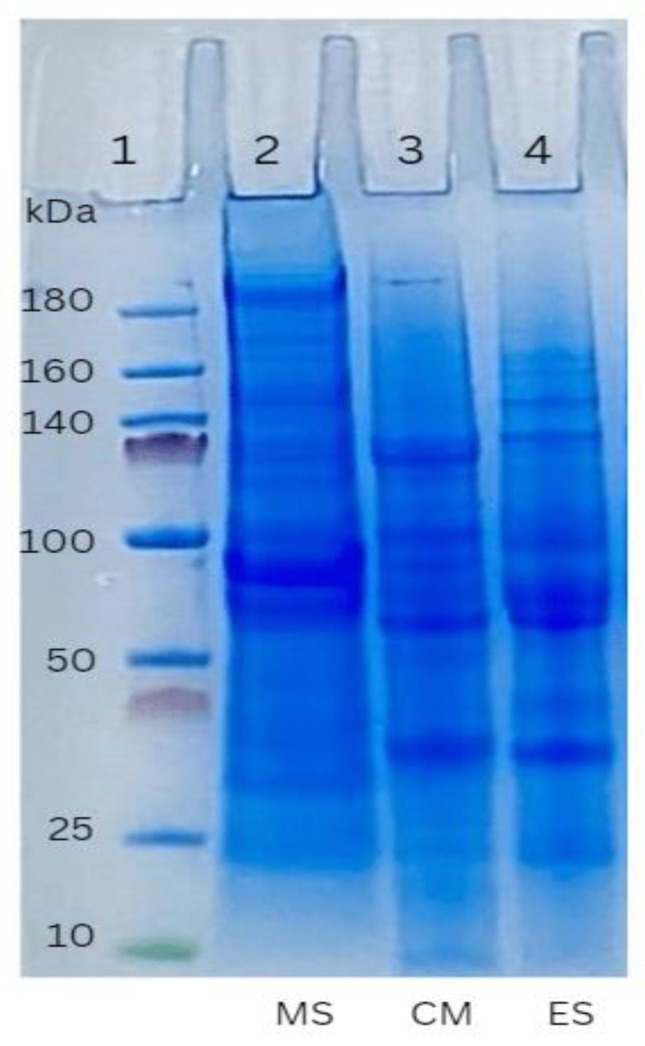
SDS-PAGE profile of extracted proteins from different sausages. MS: meat sausage; CM: commercial plant-based sausage; ES: emulsion-type sausage.

The structural protein composition of meat sausages (MS), commercial PB sausages (CM), and emulsion-type sausage (ES) was analyzed by SDS-PAGE. Figure 2 illustrates the protein profile of MS, CM, and ES. CM consisted of soy protein of more than 50% and ES consisted of grey oyster mushroom combined with chickpea (25 and 15%, respectively). The subunits of CM and ES were almost the same. According to the estimated molecular weight, the primary bands corresponded to the acidic polypeptide (AP) and basic polypeptide (BP) of glycinin (11S globulin) as well as α’-, α-, and β-subunits of β-conglycinin (7S globulin) [101]. According to the findings, there was no major difference between all three protein samples on the basis of an electrophoresis pattern. The steaming of mushrooms may denature protein and alter the molecular weight profile of the protein [102]. Albumins, globulins, glutelin-like materials, glutelins, prolamins, and prolamin-like materials were the major protein fractions in mushrooms. Gliadin and glutenin, which comprise about 85% of the proteins in wheat gluten, have particularly distinctive properties that set them apart from other plant proteins [103]. These two proteins combine with water to form the viscoelastic matrix typical of bread dough and help form the disulfide bonds that give textured plant proteins their fibrous structure [103]. Chickpea is an additional protein source included in the ES in addition to soy protein and wheat gluten. The protein pattern of legumin, which makes up 32% of the protein in chickpeas, has a protein pattern with molecular weights of 75 and 70 kDa. Legumin is larger in size and has more sulfide groups, but there is a smaller quantity of soy protein than vicilin. Despite having a small concentration, it is an essential component of the protein’s texturization due to the sulfide groups it contributes [103]. Meat sausage is different from plant-based protein patterns; the reference meat sausage has a bigger protein fraction of >180 kDa. The protein profile of protein extraction is found to identify proteins in meat sausage and plant-based sausages from electrophoresis gels that appear to have noticeably different brand characteristics due to different protein structures.

## 4. Conclusions

Grey oyster mushroom and chickpea proteins can be used as a novel alternative protein source which shows the potential to substitute up to 40% (25:15) of meat in sausages. The best mushroom-based emulsion-type sausage was developed, providing more than 36% protein, 2.25% ash, and 8.23% fat content. The products could gain acceptability from consumers regarding appearance, taste, and texture. Finally, the development of the emulsion-type sausage is expected to expand the applications of mushrooms, increase the economic value of the food industry, respond to consumer’s demands and sustainability of the future food supply, develop alternative high-protein plant-based products, and contribute to the sustainable agricultural and food industry. Further research could focus on determining the bioavailability and in vivo analysis of the emulsion-type sausages and their impact on allergenicity.

## Figures and Tables

**Table 1:** Formulations of mushroom-based emulsion-type sausages.

Formulation	GOM (%)	CF (%)	Tapioca Starch (%)	Wheat Gluten (%)	Wheat Flour (%)	Pea Protein Isolate (%)	Hydro- Colloids (%)	Canolo Oil (%)	Seasoning (%)	Water (%)
MR20	20	20	10	15	5	5	5	5	5	15
MR25	25	15	10	15	5	5	5	5	5	15
MR30	30	10	10	15	5	5	5	5	5	15

MR20: GOM to CF 20:20, *w*/*w*; MR25: GOM to CF 25:15, *w*/*w*; MR30: GOM to CF 30:10, *w*/*w*.

**Table 2:** Nutritional and functional properties of grey oyster mushroom powder and chickpea flour.

Components/Functional Properties	GOM	CF
Nutritional composition		
Moisture (%)	6.92 ± 0.10 ^b^	7.95 ± 0.50 ^a^
Ash (%)	7.15 ± 0.55 ^a^	2.72 ± 0.29 ^b^
Protein (%)	23.10 ± 0.75 ^a^	22.0.5 ± 1.55 ^b^
Fat (%)	2.52 ± 0.60 ^b^	4.61 ± 0.44 ^a^
Carbohydrate (%)	60.31	62.62
Dietary fiber (%)	55.12 ± 1.0 ^a^	10.80 ± 0.95 ^b^
Appearance	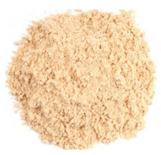	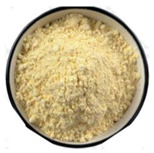
Functional properties		
Water-holding capacity (g/g)	12.75 ± 0.35 ^a^	9.16 ± 0.52 ^b^
Oil-holding capacity (mL/g)	7.82 ± 0.12 ^a^	3.97 ± 0.77 ^b^
Swelling capacity (g/g)	18.50 ± 0.25 ^a^	9.28 ± 0.35 ^b^
Emulsifying ability (%)	50.55 ± 0.75 ^a^	36.44 ± 0.25 ^b^
Emulsion stability (%)	96.10 ± 0.22 ^a^	90.15 ± 0.92 ^b^

All values are means ± SD of three replicates. Means with different superscripts in the same row are significantly different (*p <* 0.05); GOM: grey oyster mushroom powder; CF: chickpea flour.

**Table 3:** Physical and chemical properties and sensory evaluation of different emulsion-type sausages.

Formulation	C	MR20	MR25	MR30
Protein content (%)	33.23 ± 0.74 ^b^	39.87 ± 0.58 ^a^	38.07 ± 0.96 ^b^	36.95 ± 0.17 ^c^
▪ Texture profile analysis (TPA)				
Hardness (N)	4475.33 ± 470.34 ^c^	5485.79 ± 752.04 ^a^	5191 ± 1092.63 ^b^	5028.14 ± 886.52 ^b^
Springiness (mm)	0.95 ± 0.02 ^a^	0.79 ± 0.01 ^b^	0.90 ± 0.02 ^a^	0.83 ± 0.05 ^b^
Cohesiveness	0.70 ± 0.05 ^a^	0.61 ± 0.03 ^a^	0.67 ± 0.02 ^a^	0.61 ± 0.06 ^a^
Chewiness (N)	2961.37 ± 286.10 ^a^	2630.04 ± 333.26 ^a^	3124.69 ± 627.27 ^a^	3584.62 ± 734.56 ^a^
Gumminess (N)	3110.69 ± 299.01 ^a^	3321.68 ± 451.99 ^a^	3464.55 ± 727.34 ^a^	3404.29 ± 703.14 ^a^
▪ Sensory evaluation				
Overall acceptability	-	5.7 ± 1.39 ^b^	5.8 ± 1.35 ^a^	5.67 ± 1.75 ^b^
Texture	-	5.77 ± 1.79 ^a^	6.03 ± 1.55 ^a^	5.1 ± 2.06 ^a^
Taste	-	5.87 ± 1.74 ^a^	5.07 ± 2 ^a^	5.3 ± 2.03 ^a^

All values are means ± SD of three replicates; values with different superscripts in the same row are significantly different (*p <* 0.05); C: commercial; MR20: GOM to CF 20:20, *w*/*w*, MR25: GOM to CF 25:15, *w*/*w*, MR30: GOM to CF 30:10, *w*/*w*.

**Table 4:** Physical properties, cooking qualities, and sensory evaluation of emulsion-type sausages.

Formulation	KP	XG	CN
Expressible moisture (%)	0.49 ± 0.08 ^b^	0.61 ± 0.02 ^a^	0.58 ± 0.06 ^a^
▪ Emulsion stability (%) as total extrudate	2.30 ± 0.07 ^a^	4.87 ± 0.01 ^a^	3.68 ± 0.09 ^b^
▪ Purge loss (%)	3.25 ± 0.17 ^a^	5.05 ± 0.27 ^c^	4.32 ± 0.10 ^b^
▪ Cooking loss (%)	4.44 ± 0.05 ^a^	6.97 ± 0.03 ^c^	5.79 ± 0.07 ^b^
▪ Appearance	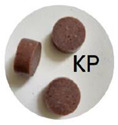	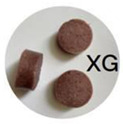	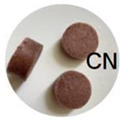
▪ Textural profile analysis (TPA)			
▪ Hardness (N)	5528.16 ± 46.45 ^a^	5462.78 ± 69.78 ^a^	5435.03 ± 34.67 ^a^
▪ Springiness (mm)	0.89 ± 0.03 ^a^	0.84 ± 0.05 ^a^	0.87 ± 0.04 ^a^
▪ Cohesiveness	0.67 ± 0.02 ^a^	0.63 ± 0.02 ^a^	0.64 ± 0.01 ^a^
▪ Chewiness (N)	3515.45 ± 0.14 ^a^	3213.63 ± 0.02 ^a^	3426.03 ± 0.02 ^a^
▪ Gumminess (N)	5340.01 ± 238.88 ^a^	5704.48 ± 321.46 ^a^	5527.8 ± 287.22 ^a^
▪ Sensory evaluation			
▪ Appearances	6.53 ± 1.80 ^a^	6.33 ± 1.97 ^a^	6.37 ± 1.63 ^a^
▪ Texture	6.23 ± 1.96 ^a^	6.03 ± 2.20 ^a^	6.1 ± 1.81 ^a^
▪ Overall	6.13 ± 1.94 ^a^	5.97 ± 2.24 ^a^	6.0 ± 1.72 ^a^
▪ Acceptance (%)	40.00	33.30	26.70

All values are means ± SD of three replicates; values with different superscripts in the same row are significantly different (*p <* 0.05). KP: konjac powder; XG: xanthan gum; CN: carrageenan.

**Table 5:** Effect of different oils on the sensory attributes of emulsion-type sausages.

Formulation	RBO	OL	CL
▪ Overall	6.47 ± 1.76 ^a^	6.17 ± 1.63 ^a^	6.43 ± 1.29 ^a^
▪ Taste	6.5 ± 2.01 ^a^	5.7 ± 1.52 ^a^	6.37 ± 1.64 ^a^
▪ Aroma	5.27 ± 2.08 ^a^	5.63 ± 1.59 ^a^	5.97 ± 1.81 ^a^
▪ Appearances	6.73 ± 1.72 ^a^	6.1 ± 1.83 ^a^	6.37 ± 1.81 ^ab^
▪ Acceptance (%)	43.30	20.00	36.70
▪ Appearance	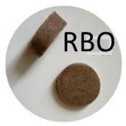	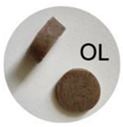	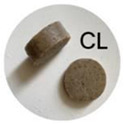

All values are means ± SD of triplicate; means with different superscripts in the same row are significantly different (*p <* 0.05); RBO: rice bran oil; OL: olive oil; CL: canola oil.

**Table 6:** Sensory determination of the developed ES and plant-based commercial sausage.

Attributes	Samples	
	ES	C
▪ Overall acceptability	6.43 ± 1.59 ^a^	6.57 ± 1.57 ^a^
▪ Texture	6.07 ± 1.68 ^a^	6.3 ± 2.07 ^a^
▪ Appearance	6.83 ± 1.60 ^a^	7.03 ± 1.64 ^a^
▪ Aroma	5.47 ± 1.57 ^ab^	6.33 ± 1.73 ^a^
▪ Taste	6.10 ± 1.70 ^a^	6.27 ± 2.05 ^a^
▪ Color	6.80 ± 1.52 ^ab^	7.00 ± 1.31 ^a^

All values are means ± SD of three replicates; values with different superscripts in the same row are significantly different (*p <* 0.05). ES: emulsion-type sausages; C: commercial plant-based sausages.

**Table 7:** Physicochemical, textural, and cooking qualities of emulsion-type sausages.

Attributes	Composition/Values	
	ES	C
Nutritional value (dry basis)		
Moisture content (%)	48.05 ±0.77 ^a^	48.85 ± 0.55 ^a^
Protein content (%)	36.92 ± 0.52 ^a^	33.23 ± 0.74 ^b^
Ash content (%)	2.25 ± 0.89 ^a^	2.40 ± 0.33 ^a^
Fat content (%)	8.23 ± 0.09 ^b^	9.79 ± 0.88 ^a^
Carbohydrate (%) by difference	4.55	5.73
Energy (cal/100 g)	239.95	243.95
Appearance (cooked sausages)	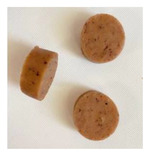	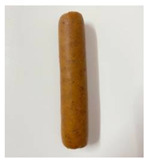
▪ Texture profile analysis (TPA)		
▪ Hardness (N)	5542.83 ± 345.00 ^a^	4475.33 ± 470.34 ^b^
▪ Springiness (mm)	0.67 ± 0.04 ^b^	0.95 ± 0.02 ^a^
▪ Cohesiveness	0.69 ± 0.02 ^a^	0.70 ± 0.03 ^a^
▪ Chewiness (N)	2963.99 ± 378.20 ^a^	2961.37 ± 286.10 ^a^
▪ Gumminess (N)	4156.07 ± 770.34 ^a^	3110.69 ± 299.01 ^b^
Color parameter (cooked sausages)		
▪ Whiteness	37.65 ± 1.45	36.22 ± 1.10
▪ ΔE	28.44 ± 0.88	27.65 ± 0.55
▪ L*	42.60 ± 0.45 ^a^	41.60 ± 0.84 ^a^
▪ a*	13.52 ± 0.33 ^a^	12.95 ± 0.59 ^a^
▪ b*	20.98 ± 0.73 ^a^	22.18 ± 0.14 ^a^
Physical properties		
▪ Cooking loss (%)	4.08 ± 1.77 ^b^	6.08 ± 1.05 ^a^
▪ Emulsion stability (%) as total extrudate	2.50 ± 0.39 ^b^	4.25 ± 0.66 ^a^
▪ Purge loss (%)	3.45 ± 0.77 ^b^	4.05 ± 0.93 ^a^

All values are means ± SD of three replicates; values with different superscripts in the same row are significantly different (*p <* 0.05). ES: emulsion-type sausages; C: commercial plant-based sausages.

## Data Availability

Data is contained within the article.

## References

[1] Choudhury D., Singh S., Seah J.S.H., Yeo D.C.L., Tan L.P. (2020). Commercialization of plant-based meat alternatives. Trends Plant Sci..

[2] Plant-Based Meat Market to Cross $15.7 Billion by 2027—Exclusive Report by Markets and Markets™. https://www.globenewswire.com/news-release/2022/12/21/2577964/0/en/Plant-based-Meat-Market-to-Cross-15-7-Billion-by-2027-Exclusive-Report-by-MarketsandMarkets.html.

[3] Hughes G.J., Ryan D.J., Mukherjea R., Schasteen C.S. (2011). Protein digestibility-corrected amino acid scores (PDCAAS) for soy protein isolates and concentrate: Criteria for evaluation. J. Agric. Food Chem..

[4] Sun C., Ge J., He J., Gan R., Fang Y. (2021). Processing, quality, safety, and acceptance of meat analogue products. Engineering.

[5] Martínez-Villaluenga C., Gulewicz P., Frias J., Gulewicz K., Vidal-Valverde C. (2008). Assessment of protein fractions of three cultivars of *Pisum sativum* L.: Effect of germination. Eur. Food Res. Tech..

[6] Chang S.T. (2006). Development of the culinary-medicinal mushrooms industry in China: Past, present and future. Int. J. Med. Mushrooms.

[7] Moore D., Chiu S.W., Pointing S.B., Hyde K.D. (2001). Filamentous fungi as food. Exploitation of Filamentous Fungi.

[8] Panuthut C. (2001). Thai Mushroom.

[9] Chirinang P., Intarapichet K.O. (2009). Amino acids and antioxidant properties of the oyster mushrooms, Pleurotus ostreatus and Pleurotus sajor-caju. Sci. Asia.

[10] Gallotti F., Lavelli V., Turchiuli C. (2020). Application of Pleurotus ostreatus β-glucans for oil–in–water emulsions encapsulation in powder. Food Hydrocoll..

[11] Jung D.Y., Lee H.J., Shin D.J., Kim C.H., Jo C. (2022). Mechanism of improving emulsion stability of emulsion-type sausage with oyster mushroom (*Pleurotus ostreatus*) powder as a phosphate replacement. Meat Sci..

[12] Boukid F. (2021). Chickpea (*Cicer arietinum* L.) protein as a prospective plant-based ingredient: A review. Int. J. Food Sci. Tech..

[13] McArdle R., Hamill R. (2011). Utilisation of hydrocolloids in processed meat systems. Processed Meats Improving Safety, Nutrition and Quality.

[14] Goff H.D., Guo Q., Spyropoulos F., Lazidis A., Norton I. (2019). The role of hydrocolloids in the development of food structure. Food Chemistry, Function and Analysis Handbook of Food Structure Development.

[15] Saha D., Bhattacharya S. (2010). Hydrocolloids as thickening and gelling agents in food: A critical review. J. Food Sci. Tech..

[16] Arora B., Kamal S., Sharma V.P. (2017). Effect of binding agents on quality characteristics of mushroom based sausage analogue. J. Food Process. Preserv..

[17] Bayram M., Bozkurt H. (2007). The use of bulgur as a meat replacement: Bulgur-sucuk (a vegetarian dry-fermented sausage). J. Sci. Food Agric..

[18] Sakai K., Sato Y., Okada M., Yamaguchi S. (2021). Improved functional properties of meat analogs by laccase catalyzed protein and pectin crosslinks. Sci. Rep..

[19] Lee E.J., Hong G.P. (2020). Effects of microbial transglutaminase and alginate on the water-binding, textural and oil absorption properties of soy patties. Food Sci. Biotechnol..

[20] Ran X., Lou X., Zheng H., Gu Q., Yang H. (2022). Improving the texture and rheological qualities of a plant-based fishball analogue by using konjac glucomannan to enhance crosslinks with soy protein. Innov. Food Sci. Emerg. Tech..

[21] Hsing Y.-l., Chern C.-G., Fan M.-J., Lu P.-C., Chen K.-T., Lo S.-F., Sun P.-K., Ho S.-L., Lee K.-W., Wang Y.-C. (2007). A rice gene activation/knockout mutant resource for high throughput functional genomics. Plant Mol. Biol..

[22] Mazlan M.M., Talib R.A., Chin N.L., Shukri R., Taip F.S., Nor M.Z.M., Abdullah N. (2020). Physical and microstructure properties of oyster mushroom-soy protein meat analog via single-screw extrusion. Foods.

[23] Bakhsh A., Lee S.-J., Lee E.-Y., Sabikun N., Hwang Y.-H., Joo S.-T. (2021). A Novel approach for tuning the physicochemical, textural, and sensory characteristics of plant-based meat analogs with different levels of methylcellulose concentration. Foods.

[24] Bakhsh A., Lee S.-J., Lee E.-Y., Hwang Y.-H., Joo S.-T. (2021). Evaluation of rheological and sensory characteristics of plant-based meat analog with comparison to beef and pork. Food Sci. Anim. Resour..

[25] Ali A., Devarajan S. (2017). Nutritional and Health Benefits of Rice Bran Oil.

[26] Lai O.-M., Jacoby J.J., Leong W.-F., Lai W.-T., Cheong L.-Z., Xu X. (2019). Chapter 2—Nutritional studies of rice bran oil. Rice Bran and Rice Bran Oil.

[27] Ghosh S.K., Pal T. (2007). Interparticle coupling effect on the surface plasmon resonance of gold nanoparticles: From theory to applications. Chem. Rev..

[28] Putnik P., Kovačević D.B. (2021). Meat consumption: Theory, practice and future prospects. Theor. Prac. Meat Proc..

[29] Peng W., Xu X.L., Zhou G.H. (2009). Effects of meat and phosphate level on water-holding capacity and texture of emulsion-type sausage during storage. Agric. Sci. China.

[30] Zinia S.A., Rahim A., Jony A.L., Begum A.A., Mazumder M.A.R. (2019). The roles of okara powder on the processing and nutrient content of roti and paratha. Int. J. Agric. Environ. Sci..

[31] AOAC International, Official Method 985.29 (2019). Total Dietary Fiber in Foods.

[32] Mesías M., Morales F.J. (2017). Effect of different flours on the formation of hydroxymethylfurfural, furfural, and dicarbonyl compounds in heated glucose/flour systems. Foods.

[33] Sosulski F.W., Humbert E., Bui S.K., Jones J.O. (1996). Functional properties of rapeseed flour concentrates and isolate. J. Food Sci..

[34] Elaveniya E., Jayamuthungai J. (2014). Functional, physicochemical and antioxidant properties of dehydrated banana blossom powder and its incorporation in biscuits. Int. J. Chem. Tech. Res..

[35] Sathe S.K., Deshpande S.S., Salunkhe D.K. (1983). Functional properties of black gram (*Phaseolus mungo* L.) proteins. Lebensm.-Wiss. Technol..

[36] Aguirre M.E., Owens C.M., Miller R.K., Alvarado C.Z. (2018). Descriptive sensory and instrumental texture profile analysis of woody breast in marinated chicken. Poult. Sci..

[37] Wang X., Wang S.J., Nan Y., Liu G.Q. (2020). The effects of oil type and crystallization temperature on the physical properties of vitamin C-loaded oleogels prepared by an emulsion-templated approach. Food Func..

[38] Hughes E., Cofrades S., Troy D.J. (1997). Effect of fat level, oat fiber and carrageenan on frankfurters formulated with 5, 12 and 30% fat. Meat Sci..

[39] Cheetangdee N. (2017). Characteristics of sausages as influenced by partial replacement of pork back-fat using pre-emulsified soybean oil stabilized by fish proteins isolate. Agric. Nat. Resour..

[40] Lee J.-S., Oh H., Choi I., Yoon C.S., Han J. (2022). Physico-chemical characteristics of rice protein-based novel textured vegetable proteins as meat analogues produced by low-moisture extrusion cooking technology. LWT.

[41] AOAC International, Official Method 950.46 (2019). Moisture in Meat and Meat Products.

[42] AOAC International, Official Method 920.153 (2019). Ash in Meat and Meat Products.

[43] AOAC International, Official Method 981.10 (2019). Crude Protein in Meat and Meat Products.

[44] AOAC International, Official Method 922.06 (2019). Fat in Grain and Flour.

[45] Food and Agriculture Organization Food Energy—Methods of Analysis and Conversion Factors. https://www.fao.org/uploads/media/FAO_2003_Food_Energy_02.pdf.

[46] Wichchukit S., O’Mahony M. (2015). The 9-point hedonic scale and hedonic ranking in food science: Some reappraisals and alternatives. J. Sci. Food Agric..

[47] Lopera-Cardona S., Gallardo C., Umañ-Gallego J., Gil L.M. (2016). Comparative study of the physicochemical, compositional and functional properties of eight flours obtained from different plant materials found in Colombia. Food Sci. Tech. Int..

[48] Raghavendra S.N., Ramachandra Swamy S.R., Rastogi N.K., Raghavarao K.S.M.S., Kumar S., Tharanathan R.N. (2006). Grinding characteristics and hydration properties of coconut residue: A source of dietary fiber. J. Food Eng..

[49] Yegrem L., Mengestu D., Legesse O., Abebe W., Girma N. (2022). Nutritional compositions and functional properties of New Ethiopian chickpea varieties: Effects of variety, grown environment and season. Int. J. Food Proper..

[50] Grigelmo-Miguel N., Martín-Belloso O. (1999). Comparison of dietary fibre from by-products of processing fruits and greens and from cereals. LWT-Food Sci. Tech..

[51] Lan G., Chen H., Chen S., Tian J. (2012). Chemical composition and physicochemical properties of dietary fiber from Polygonatum odoratum as affected by different processing methods. Food Res. Int..

[52] Guillon F., Champ M. (2000). Structural and physical properties of dietary fibres, and consequences of processing on human physiology. Food Res. Int..

[53] Viuda-Martos M., Ruiz-Navajas Y., Martin-Sánchez A., Sánchez-Zapata E., Fernández-López J., Sendra E., Sayas-Barberá E., Navarro C., Pérez-Álvarez J.A. (2012). Chemical, physico-chemical and functional properties of pomegranate (*Punica granatum* L.) bagasses powder coproduct. J. Food Eng..

[54] Kaushal P., Kumar V., Sharma H.K. (2012). Comparative study of physicochemical, functional, anti-nutritional and pasting properties of taro (*Colocasia Esculenta*), rice (*Oryza Sativa*), pegion pea (*Cajanus Cajan*) flour and their Blends. LWT Food Sci. Technol..

[55] Khalifa G.K., Eljack A.E., Mohammed M.I., Elamin O.M., Mohamed E.S. (2013). Yield stability in common bean genotypes (*Phaseolus Vulgaris* L.) in the Sudan. J. Plant Breed. Crop Sci..

[56] Khan T.N., Meldrum A., Croser J.S. (2016). Pea: Overview.

[57] Drummen M., Tischmann L., Gatta-Cherifi B., Adam T., Westerterp-Plantenga M. (2018). Dietary protein and energy balance in relation to obesity and co-morbidities. Front. Endocrinol..

[58] Institute of Medicine, Food and Nutrition Board (1997). Dietary Reference Intakes for Calcium, Phosphorus, Magnesium, Witamin D, and Fluorid.

[59] Verma M.M., Ledward D.A., Lawrie R.A. (1984). Utilization of chickpea flour in sausages. Meat Sci..

[60] Kilinççeker O., Kurt Ş. (2010). Effects of chickpea (*Cicer arietinum*) flour on quality of deep-fat fried chicken nugget. J. Food Agric. Environ..

[61] Verma A.K., Banarjee R., Sharma B.D. (2012). Quality of low fat chicken nuggets: Effect of sodium chloride replacement and added chickpea (*Cicer arietinum* L.) hull flour. Asian-Aust. J. Anim. Sci..

[62] Jukantil A.K., Gaur P.M., Gowda C.L.L., Chibbar R.N. (2012). Nutritional quality and health benefits of chickpea (*Cicer arietinum* L.): A review. Br. J. Nutr..

[63] Webb D., Plattner B.J., Donald E., Funk D., Plattner B.S., Alavi S. (2020). Role of chickpea flour in texturization of extruded pea protein. J. Food Sci..

[64] Why You Should Buy a Bag of Chickpea Flour. https://www.epicurious.com/ingredients/how-to-cook-and-bake-with-chickpea-flour-article.

[65] Sharima-Abdullah N., Hassan C.Z., Arifin N., Huda-Faujan N. (2018). Physicochemical propertiesand consumer preference of imitation chickennuggets produced from chickpea flour and texturedvegetable protein. Int. Food Res. J..

[66] Osen R., Toelstede S., Eisner P., Schweiggert-Weisz U. (2015). Effect of high moisture extrusion cooking on protein-protein interactions of pea (*Pisum sativum* L.) protein isolates. Int. J. Food Sci. Tech..

[67] Mohammed I., Ahmed A.R., Senge B. (2012). Dough rheology and bread quality of wheat-chickpea flour blends. Ind. Crop. Prod..

[68] Ismail Yılmaz I., Orhan Dağlıoğlu O. (2003). The effect of replacing fat with oat bran on fatty acid composition and physicochemical properties of meatballs. Meat Sci..

[69] Sarıçoban C., Yılmaz M.T., Karakaya M. (2009). Response surface methodology study on the optimization of effects of fat, wheat bran and salt onchemical, textural and sensory properties of patties. Meat Sci..

[70] Wan Rosli W.I., Solihah M.A., Aishah M., NikFakurudin N.A., Mohsin S.S.J. (2011). Colour, textural properties, cooking characteristics and fibre content of chicken patty added with oyster mushroom (*Pleurotus sajor-caju*). Int. Food Res. J..

[71] Ding Y. (2005). Process for Creating a Thermo-Irreversible Konjac Gel Food. U.S. Patent.

[72] Candogan K., Kolsarici N. (2003). The effects of carrageenan and pectin on some quality characteristics of low fat beef frankfurters. Meat Sci..

[73] Shand P.J., Sofos J.N., Schmidt G.R. (1994). Kappa-carrageenan, sodium chloride and temperature affect yield and texture of structured beef Rolls. J. Food Sci..

[74] Han M., Bertram C. (2017). Designing healthier comminuted meat products: Effect of dietary fibers on water distribution and texture of a fat-reduced meat model system. Meat Sci..

[75] Cornet S.H.V., Edwards D., van der Goot A.J., van der Sman R.G.M. (2020). Water release kinetics from soy protein gels and meat analogues as studied with confined compression. Innov. Food Sci. Emerg. Technol..

[76] Schreuders F.K.G., Dekkers B.L., Bodnár I., Erni P., Boom R.M., Jan A., Goot V.D. (2019). Comparing structuring potential of pea and soy protein with gluten for meat analogue preparation. J. Food Eng..

[77] Wi G., Bae J., Kim H., Cho Y., Choi M.-J. (2020). Evaluation of the Physicochemical and Structural Properties and the Sensory Characteristics of Meat Analogues Prepared with Various Non-Animal Based Liquid Additives. Foods.

[78] Pietrasik Z., Duda Z. (2000). Effect of fat content and soy protein/carragenan mix on the quality characteristics of comminuted, scalded sausages. Meat Sci..

[79] Carballo J., Barreto G., Colmenero F.J. (1995). Starch and egg white influence on properties of bologna sausage as related to fat content. J. Food Sci..

[80] Grigelmo-Miguel N., Martin-Belloso O. (1997). Peach dietary fiber as a food Ingredient. IFT Book Abstr..

[81] Grigelmo-Miguel N., Abadias-Seros M.I., Martin-Belloso O. (1999). Characterization of low-fat high dietary fibre frankfurters. Meat Sci..

[82] Egbert R.W., Huffman D.L., Chen C., Dylewski D.P. (1991). Development of low-fat ground beef. Food Technol..

[83] Candogan K., Kolsarici N. (2003). Storage stability of low-fat beef frankfurters formulated with carrageenan or carrageenan with pectin. Meat Sci..

[84] Traynham T.L., Myers D.J., Carriquiry A.L., Johnson L.A. (2007). Evaluation of water-holding capacity for wheat-soy flour blends. J. Am. Oil Chem. Soc..

[85] Wang Z., Liang J., Jiang L., Li Y., Wang J., Zhang H., Li D., Han F., Li Q., Wang R. (2015). Effect of the interaction between myofibrillar protein and heat-induced soy protein isolates on gel properties. CyTA J. Food.

[86] Cornet S.H., Snel S.J., Lesschen J., van der Goot A.J., van der Sman R.G. (2021). Enhancing the water holding capacity of model meat analogues through marinade composition. J. Food Eng..

[87] Lin K.W., Keeton J.T. (1998). Textural and physicochemical properties of low-fat, precooked ground beef patties containing carrageenan and sodium alginate. J. Food Sci..

[88] Kang Y., Siegel P.M., Shu W., Drobnjak M., Kakonen S.M., Cordón-Cardo C., Guise T.A., Massagué J. (2003). A multigenic program mediating breast cancer metastasis to bone. Cancer Cell.

[89] Dai F.C., Lee C.F., Ngai Y.Y. (2002). Landslide risk assessment and management: An overview. Eng. Geol..

[90] Draget K.I., Bræk G.S., Smidsrød O. (1994). Alginic acid gels: The effect of alginate chemical composition and molecular weight. Carbohydr. Polym..

[91] Park J., Park J., Jang S., Kim S., Kong S., Choi J., Ahn K., Kim J., Lee S., KIm S. (2008). FTFD: An informatics pipeline supporting phylogenomic analysis of fungal transcription factors. Bioinformatics.

[92] Nair M.S., Tomar M., Punia S., Kukula-Koch W., Kumar M. (2020). Enhancing the functionality of chitosan-and alginate-based active edible coatings/films for the preservation of fruits and vegetables: A review. Int. J. Biol. Macromol..

[93] Selani M.M., Bianchini A., Ratnayake W.S., Flores R.A., Massarioli A.P., de Alencar S.M., Canniatti Brazaca S.G. (2016). Physicochemical, functional and antioxidant properties of tropical fruits co-products. Plant Foods Hum. Nutr..

[94] Ahmad H., Chohan T.A., Mudassir J., Mehta P., Yousef B., Zaman A., Ali A., Qutachi O., Chang M.-W., Fatouros D. (2022). Evaluation of sustained-release in-situ injectable gels, containing naproxen sodium, using in vitro, in silico and in vivo analysis. Int. J. Pharm..

[95] Cîrstea (Lazăr) N., Nour V., Boruzi A.I. (2023). Effects of Pork Backfat Replacement with Emulsion Gels Formulated with a Mixture of Olive, Chia and Algae Oils on the Quality Attributes of Pork Patties. Foods.

[96] Kim G.D., Seo J.K., Yum H.W., Jeong J.Y., Yang H.S. (2017). Protein markers for discrimination of meat species in raw beef, pork and poultry and their mixtures. Food Chem..

[97] Zeraatkar D., Han M.A., Guyatt G.H., Vernooij R.W., El Dib R., Cheung K., Milio K., Zworth M., Bartoszko J., Valli C. (2019). Red and processed meat consumption and risk for all-cause mortality and cardiometabolic outcomes: A systematic review and meta-analysis of cohort studies. Ann. Int. Medic..

[98] Giangiaconmo R., Messina G. (1988). Determinazione oggetiva del colore del latte alimentare mediante colorimetria tristimolo. Sci. E Tec. Latt.-Casearia.

[99] Petracci M., Mudalal S., Bonfiglio A., Cavani C. (2013). Occurrence of white striping under commercial conditions and its impact on breast meat quality in broiler chickens. Poult. Sci..

[100] Botella-Martínez C., Viuda-Martos M., Fernández-López J.A., Pérez-Alvarez J.A., Fernández-López J. (2022). Development of plant-based burgers using gelled emulsions as fat source and beetroot juice as colorant: Effects on chemical, physicochemical, appearance and sensory characteristics. LWT.

[101] Webb D., Li Y., Alavi S. (2023). Chemical and physicochemical features of common plant proteins and their extrudates for use in plant-based meat. Trends Food Sci. Technol..

[102] Kyriakopoulou K., Dekkers B., van der Goot A.J., Galanakis C.M. (2019). Chapter 6—Plant-based meat analogues. Sustainable Meat Production and Processing.

[103] Samard S., Ryu G.-H. (2019). Physicochemical and functional characteristics of plant protein-based meat analogs. J. Food Proc. Preserv..

